# Supplementary material for: Seed choice in ground beetles is driven by surface-derived hydrocarbons
Source: Commun Biol. 2022 Jul 21;5:724. doi: 10.1038/s42003-022-03678-1 (PMC9304415; doi:10.1038/s42003-022-03678-1)
Supplement: Supplementary file 2 — Reporting summary [file 42003_2022_3678_MOESM2_ESM.pdf]

nature portfolio

Corresponding author(s): Khaldoun Ali

Last updated by author(s): Jun 7, 2022

Reporting Summary

Nature Portfolio wishes to improve the reproducibility of the work that we publish. This form provides structure for consistency and transparency in reporting. For further information on Nature Portfolio policies, see our Editorial Policies and the Editorial Policy Checklist.

Statistics

For all statistical analyses, confirm that the following items are present in the figure legend, table legend, main text, or Methods section.

n/a	Confirmed	
☐	☒	The exact sample size (n) for each experimental group/condition, given as a discrete number and unit of measurement
☐	☒	A statement on whether measurements were taken from distinct samples or whether the same sample was measured repeatedly
☐	☒	The statistical test(s) used AND whether they are one- or two-sided<br>*Only common tests should be described solely by name; describe more complex techniques in the Methods section.*
☒	☐	A description of all covariates tested
☐	☒	A description of any assumptions or corrections, such as tests of normality and adjustment for multiple comparisons
☐	☒	A full description of the statistical parameters including central tendency (e.g. means) or other basic estimates (e.g. regression coefficient) AND variation (e.g. standard deviation) or associated estimates of uncertainty (e.g. confidence intervals)
☐	☒	For null hypothesis testing, the test statistic (e.g. F, t, r) with confidence intervals, effect sizes, degrees of freedom and P value noted<br>*Give P values as exact values whenever suitable.*
☒	☐	For Bayesian analysis, information on the choice of priors and Markov chain Monte Carlo settings
☐	☒	For hierarchical and complex designs, identification of the appropriate level for tests and full reporting of outcomes
☒	☐	Estimates of effect sizes (e.g. Cohen's d, Pearson's r), indicating how they were calculated

Our web collection on statistics for biologists contains articles on many of the points above.

Software and code

Policy information about availability of computer code

Data collection	Data were collected manually and no data collection software was used.
Data analysis	R v.4.0.3 (R Development Team 2020) was used for all data analysis.

For manuscripts utilizing custom algorithms or software that are central to the research but not yet described in published literature, software must be made available to editors and reviewers. We strongly encourage code deposition in a community repository (e.g. GitHub). See the Nature Portfolio guidelines for submitting code & software for further information.

Data

Policy information about availability of data

All manuscripts must include a data availability statement. This statement should provide the following information, where applicable:

- Accession codes, unique identifiers, or web links for publicly available datasets
- A description of any restrictions on data availability
- For clinical datasets or third party data, please ensure that the statement adheres to our policy

Datasets analyzed and presented in this study are currently available from the corresponding author upon request. Data sets will be uploaded to a data repository if and this statement will be updated once data are published and an accession number or DOI is provided.

Field-specific reporting

Please select the one below that is the best fit for your research. If you are not sure, read the appropriate sections before making your selection.

☐ Life sciences ☐ Behavioural & social sciences ☒ Ecological, evolutionary & environmental sciences

For a reference copy of the document with all sections, see nature.com/documents/nr-reporting-summary-flat.pdf

Ecological, evolutionary & environmental sciences study design

All studies must disclose on these points even when the disclosure is negative.

Study description	The study looks into the the ecology of omnivorous carabid beetles and describe aspects of the sensory and behavioral ecology involved in seed feeding of Poecilus corvus, Pterostichus melanarius, Harpalus amputatus, and Amara littoralis (Coleoptera: Carabidae)
Research sample	Seeds of three different brassicaceous species (Brassicaceae: Brassica napus L., Sinapis arvensis L., Thlaspi arvense L.) were used as model species in this study. Seeds of these three species arenall considered high in lipids, and all are weeds of considerable importance in arable fields of the Northern Great Plains region of North America.<br>Adults of the omnivorous carabid species Poecilus corvus (Leconte), Harpalus amputatus Say, Pterostichus melanarius (Illiger), and Amara littoralis Dejean which are known to consume weed seeds, were used in this study.
Sampling strategy	Samples of seed species were hand-collected from different field sites at the Kernen Crop Research Farm near Saskatoon, SK, Canada. Seed samples were stored at 5 C until use. Live adults of the carabid species were collected from different field sites at the Kernen Crop Research Farm in the summers of 2018-20 via dry pitfall trapping. Seed species were offered to carabid predators in three-choice multiple choice Petri-dish feeding arenas. Twenty five seeds were used per each weed species in the seed feeding experiments. The number or replicates varied across the experiments described in the manuscript depending on the experimental design. There were enough replicates in the experiment to endow the statistical models with sufficient predictive power.
Data collection	The number of the seeds consumed by the carabid predators were manually recored in the seed-feeding experiments. TThe TSS Utility software with a link to the NSIT Library was used for analyzing the chromatograms and identifying the seed volatile chemicals in GC-MS analyses. KAA (the lead author) conducted all the experiments and collected and analyzed all the data.
Timing and spatial scale	The experiments were carried out at three consecutive summers in Saskatoon, SK, Canada. The study started at the Spring of 2018 and lasted until th end of Summer of 2020.
Data exclusions	No data were excluded form the analysis.
Reproducibility	Each and every experiment described in the manuscript was carries out over two seasons (two years) and reolicated multiple times. Chemical extraction were replicated five times per each seed species. Behavioral experiments were replicated 10 times across treatments and control. Seed feeding experiments were repeated 15 or 25 times across treatments and control. These high levels of replication ensured that results were reproducible and predictive power of the statistical models was high and reliable.
Randomization	Randomization of sample choice and treatment assignments were randomized across the experiments. These procedures are described in the manuscript.
Blinding	Blinding was not relevant to this study.

Did the study involve field work? ☒ Yes ☐ No

Field work, collection and transport

Field conditions	Temperature was around an average of 15 C (60 F) in May and early June and hovered around mid-30s C (90-95 F) in July and August. Average rainfall was around 130 mm.
Location	Kernen Crop Research Farm near Saskatoon, SK, Canada (52°09’10.3” N 106°32’41.5” W).
Access & import/export	Kernen Crop Research Farm is a research center managed by the Plant Sciences Department at the University of Saskatchewan. Therefore, no special permissions were needed to access field sites for seed and insect samplings.
Disturbance	There was no disturbance to be reported for this study.

Reporting for specific materials, systems and methods

We require information from authors about some types of materials, experimental systems and methods used in many studies. Here, indicate whether each material, system or method listed is relevant to your study. If you are not sure if a list item applies to your research, read the appropriate section before selecting a response.

Materials & experimental systems

n/a	Involved in the study
☒	☐ Antibodies
☒	☐ Eukaryotic cell lines
☒	☐ Palaeontology and archaeology
☐	☒ Animals and other organisms
☒	☐ Human research participants
☒	☐ Clinical data
☒	☐ Dual use research of concern

Methods

n/a	Involved in the study
☒	☐ ChIP-seq
☒	☐ Flow cytometry
☒	☐ MRI-based neuroimaging

Animals and other organisms

Policy information about studies involving animals; ARRIVE guidelines recommended for reporting animal research

Laboratory animals	Poecilus corvus, Harpalus amputatus, Pterostichus melanarius, and Amara littoralis
Wild animals	No wild animals were observed or experimented on for the purposes of this study.
Field-collected samples	The experimental insects were collected via dry pitfall traps. Pitfall traps were enclosed into cages of fine wire mesh (σ = 1.1 cm) to prevent vertebrates from entering the traps and ravaging the catches. Traps were emptied every three days and the collected insects were placed into plastic boxes (40 cm × 25 cm, 25 cm depth) lined with plant material and moist filter paper then brought to the lab for identification and experimentation.
Ethics oversight	The experimental work was carried out on insect species. Therefore, no ethics approvals were required to carry out the experiments. The experimental protocols followed the ethical standards described in published studies of insect sensory, chemical, and behavioral ecology.

Note that full information on the approval of the study protocol must also be provided in the manuscript.
